# HIV transmission and pre-exposure prophylaxis in a high risk MSM population: A simulation study of location-based selection of sexual partners

**DOI:** 10.1371/journal.pone.0189002

**Published:** 2017-11-30

**Authors:** Olivier Robineau, Annie Velter, Francis Barin, Pierre-Yves Boelle

**Affiliations:** 1 Sorbonne Universités - Univ Paris 06, INSERM, Institut Pierre Louis d’Epidémiologie et de Santé Publique, INSERM U1136, Paris, France; 2 Service Universitaire des Maladies Infectieuses et du Voyageur, Centre Hospitalier Gustave Dron, Tourcoing, France; 3 Département des maladies infectieuses, Univ Lille 2, Lille, France; 4 Santé Publique France, Saint Maurice, France; 5 Université François-Rabelais, INSERM UMR966, Tours, France; 6 Centre Hospitalier Régional Universitaire, Centre National de Référence du VIH, Tours, France; Agencia de Salut Publica de Barcelona, SPAIN

## Abstract

**Objective:**

In France, indications for pre-exposure prophylaxis (PrEP) for HIV prevention are based on individual-level risk factors for HIV infection. However, the risk of HIV infection may also depend on characteristics of sexual partnerships. Here we study how place-based selection of partners change transmission and the overall efficiency of PrEP.

**Methods:**

We used the PREVAGAY survey of sexual behavior and HIV serostatus in men who have sex with men (MSM) in a Parisian district to look for associations between sexual network characteristics and HIV infection. We then simulated HIV transmission in a high-risk MSM population. We used information about venues visited to meet casual sexual partners (clubs, backrooms or saunas) to define sexual networks. We then simulated HIV transmission in these networks and assessed the impact of PrEP in this population.

**Results:**

In the PREVAGAY study, we found that HIV serostatus changed with the type of venues visited, in addition to other individual risk factors. In simulations, we found similar differences in HIV incidence when the choice of venues visited was not random. The use of PrEP allowed reducing incidence, irrespective of the venues visited by PrEP users. However, with the same amount of PrEP, the number of infections adverted could almost double depending on network structure and venues visited by PrEP users.

**Conclusion:**

This study shows that characteristics of the sexual network structure can strongly impact the effectiveness of PrEP interventions. These should be considered further to refine individual risk assessment and maximize the effect of individual-based prevention policies.

## Introduction

In Western countries, HIV incidence and prevalence remains the largest in men who have sex with men (MSM) [1]. Differences in risk factors for HIV infection [2] contribute to the variability in the reported ranges [3–5]. For example, in a Parisian district where the prevalence of HIV infection was 18% in MSM attending gay venues [6], HIV infection incidence was 4-times higher than the overall incidence in French MSM [7,8]. In addition to differences in individual risk factors, the network of sexual contacts may change the probability of infection by HIV [9,10]. A better understanding of how sexual network structure changes HIV incidence is all the more required as new prevention approaches target all individuals whose personal characteristics put them at high risk. For example, in Western countries, the highly effective pre-exposure prophylaxis (PrEP) [11–13] is prescribed to MSM with a HIV-positive sexual partner *or* recent bacterial STI *or* a high number of sex partners *or* a history of inconsistent condom use *or* in commercial sex workers [14,15]. Individual risk factors are thus already common in this population, making sexual network characteristics increasingly relevant to explain variation in impact.

Place-based social mixing are of importance for communicable disease spread [16]. The type of venues attended by MSM structures the sexual contact network, determining the pool of possible partners and the type of sexual practice [17,18]. Indeed, large variation in the prevalence of HIV has been reported according to venues attended [19–22].

Here, we aimed at evaluating how the venues visited may affect HIV spread and prevention through differences in the network structure. We first examined the correlation between venues visited and HIV serostatus in the PREVAGAY study, including French MSM attending gay venues in a Parisian district. Then, we used simulations to determine the effect of assortativity in venues attended on HIV dissemination. We then studied whether the population effect of PrEP changed according to venues visited by PrEP users.

## Material and method

The PREVAGAY survey provided data on HIV serostatus and places visited by MSM (see *Data*). Sexual networks were simulated based on these data (see *Dynamic sexual network modelling*) and under different assumptions for network characteristics that might be of interest in the epidemic spread (see *Allocating partnerships to venues* and *Effect of correlation between venues visited*). The spread of HIV was simulated in these networks (see *HIV progression*, *transmission*, *screening and interventions*). Finally, we compared the effect of PrEP under different scenarios (see *Simulation Scenarios*).

### Data

The PREVAGAY survey was conducted in commercial gay venues in Paris in 2009 [6]. MSM were eligible for the survey if they were at least 18 years old, had had sex with men in the previous 12 months, and could read and speak French quite well. Eight hundred eighty-six (886) MSM reported socio-demographic data and sexual behavior. They categorized the venues they attended as “bar/clubs without backrooms” (clubs), “saunas with sex” (saunas) and “backrooms/darkrooms/videoclubs/sexclubs” (backrooms). For each participant, a blood sample was obtained concomitantly to test for HIV infection.

We used logistic regression to analyze HIV serostatus (positive/negative) according to individual MSM characteristics including number of partners, risky anal intercourse, age and places visited to meet sexual partners. A P-value less than 0.05 was considered as statistically significant. Analyses were conducted with the R software [23]. S1 Appendix is a data file including variables used in this analysis.

### Dynamic sexual network modelling

**Fig 1:**
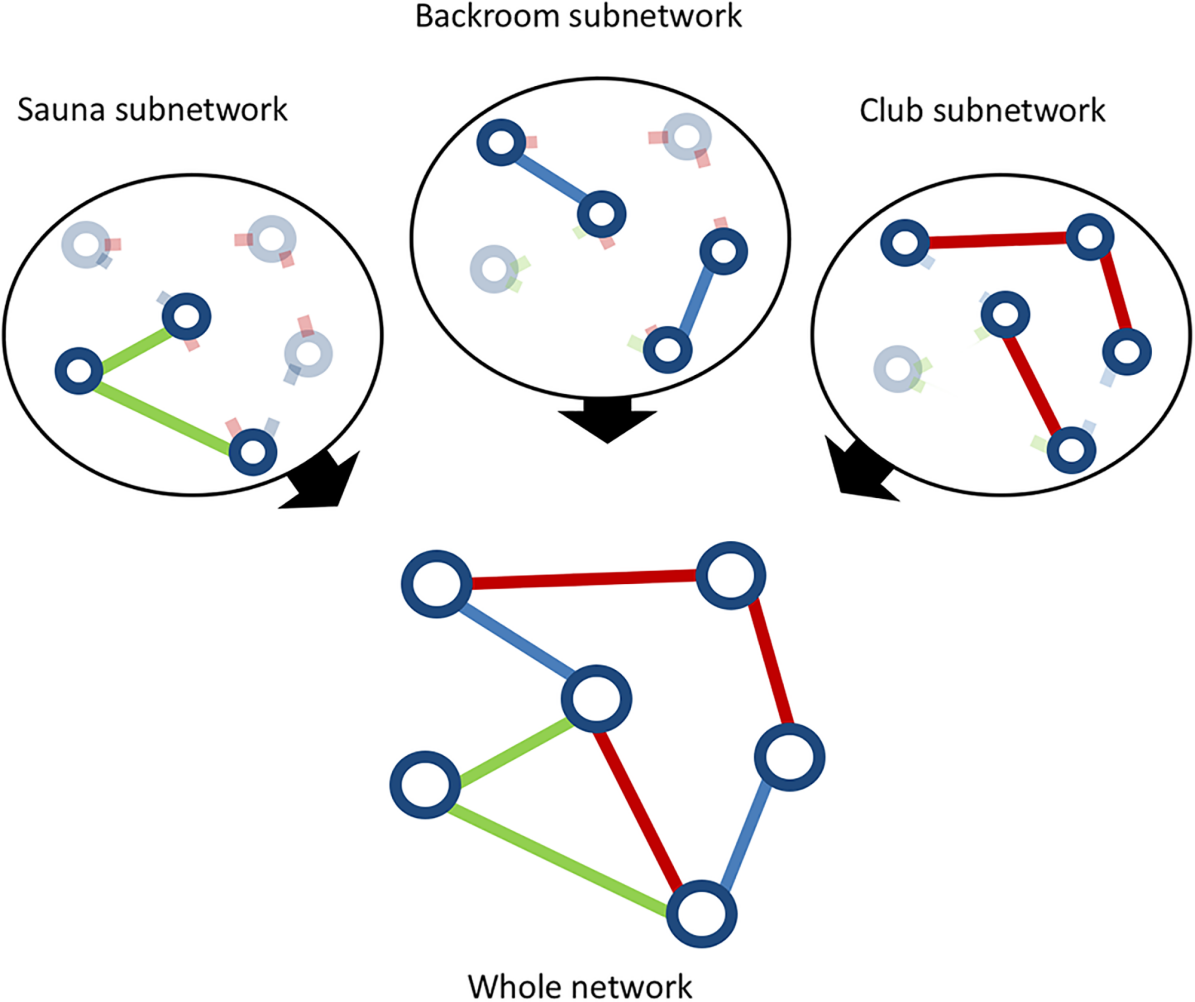
Principle for network construction. Each venue type is modelled using an ST-ERGM as a separate layer (Sauna subnetwork, Backroom subnetwork and Club subnetwork). Subnetworks are overlaid to obtain the whole network.

Casual sexual partnerships are short-lived and changing over time. This can be described by “dynamic stochastic networks”, where individuals are “nodes” and “sexual partnerships” are edges changing with time. Here, we described sexual partnerships in three layers corresponding to venue types (clubs, saunas, backrooms) and combined those in a final network (Fig 1). We simulated a virtual population of 10000 MSM by sampling with replacement from the original PREVAGAY participants so that the venues visited matched the PREVAGAY data. Then, we used a separable temporal exponential random graph model (ST-ERGM) [24–28] to describe the creation and dissolution of casual sexual partnerships in each venue type. We assumed a rate of dissolution 0.8 day^-1^, so that 80% partnerships ending in less than 2 days. The rate of new partnerships creation was chosen to balance dissolutions, so that the average number of partnerships in the population remained constant over time. The R package statnet was used to simulate ST-ERGM [29–32].

### Allocating partnerships to venues

In the PREVAGAY study, participants reported an overall number of casual partners, but not the place where they met. Therefore, we explored three rules for allocating new partnerships to venues. Rule “EQL” corresponded to allocating partnerships equally between the three venue types; Rule “BKR” corresponded to twice more partnerships in backrooms than elsewhere; and rule “DNS” allocated partnerships with equal density to all venue types (Table 1)–In a network, density is the percentage of actual partnerships (edges) out of the maximum number of potential partnerships.

**Table 1:** Rules for allocating partnerships according to venues visited. Density is computed relative to the total number of potential partnerships.

Partnership allocation rule	Description	Proportion of partnerships allocated to each venue type (%) (*Density of contacts*, *per million potential partnerships*)		
		Club	Backroom	Sauna
DNS	equal density	42% *(0*.*041)*	16% *(0*.*041)*	42% *(0*.*041)*
EQL	equal number	33% *(0*.*033)*	33% *(0*.*084)*	33% *(0*.*033)*
BKR	double in BKR	25% *(0*.*025)*	50% *(0*.*126)*	25% *(0*.*025)*

### Effect of correlation between venues visited

We investigated the effect of correlation between venues attended on the cumulated incidence of HIV infection and on the proportion of infection adverted in scenarios with PrEP. To this end, we compared simulations in networks based on the original PREVAGAY data with simulations in networks where each venue type was attended independently. The latter networks, denoted RND, were built by permuting venues attended between individuals at random. Partnerships were allocated using the 3 rules mentioned above, leading to RND/DNS, RND/EQL and RND/BKR simulations.

### HIV progression, transmission, screening and interventions

The model for HIV infection history and transmission was similar to previous descriptions [26,33–35]. Without biomedical intervention (PrEP *or* treatment), viral load after infection followed one of three different patterns. Each pattern presented a peak of viral load during the acute phase of infection, then a slow decrease followed by a rise during late stage of infection. In the simulations, viral load was updated monthly according to pre-specified probabilities. With HAART, individuals could be good responder (83% of the cases), partial responder (8%) and non-responder (9%) and the viral load changed accordingly [36]. Transmission occurred between individuals when a HIV infected person was linked to a non-infected person in the sexual network. The probability of transmission was computed as a function of viral load, modified by interventions (condom use, treatment, PrEP). Quantitative details are given in the S2 Appendix. The baseline scenario for simulation implemented annual screening of all individuals who did not know their HIV status. Highly active anti-retroviral therapy (HAART) was started in the month following a first positive screening test as recommended by international guidelines (treatment as prevention (TasP)) [37]. Condom use was as described in the PREVAGAY study (65%, 23%, 12% of individuals using condom always, inconstantly or never during anal intercourse). In scenario with PrEP, HIV screening frequency was increased to every three months in those receiving PrEP, but no other parameters were modified.

### Simulation scenarios

Simulations started with 300 infected individuals chosen at random and ran without PrEP intervention until HIV prevalence reached approximately 18%, similar to the PREVAGAY situation (we verified that the results were little affected by this choice). Then, we introduced PrEP (0%, 10% and 20% of coverage) and investigated PrEP use according to venue types visited. Scenarios were labelled “homogeneous” when PrEP was used irrespective of the venues visited, and “heterogeneous” when PrEP adopters were those visiting a particular venue type. We computed incidence and the proportion of infections adverted over the 36 months following PrEP introduction, relative to the baseline scenario (no PrEP). Finally, we computed the direct effect of PrEP by incidence reduction in individuals belonging to the subpopulation in which PrEP was used (e.g: individuals visiting saunas, clubs or backrooms), and the indirect effect as incidence reduction in other individuals. In all cases, results were averaged over 120 simulations.

## Results

### Venues visited and HIV serostatus in PREVAGAY

**Fig 2:**
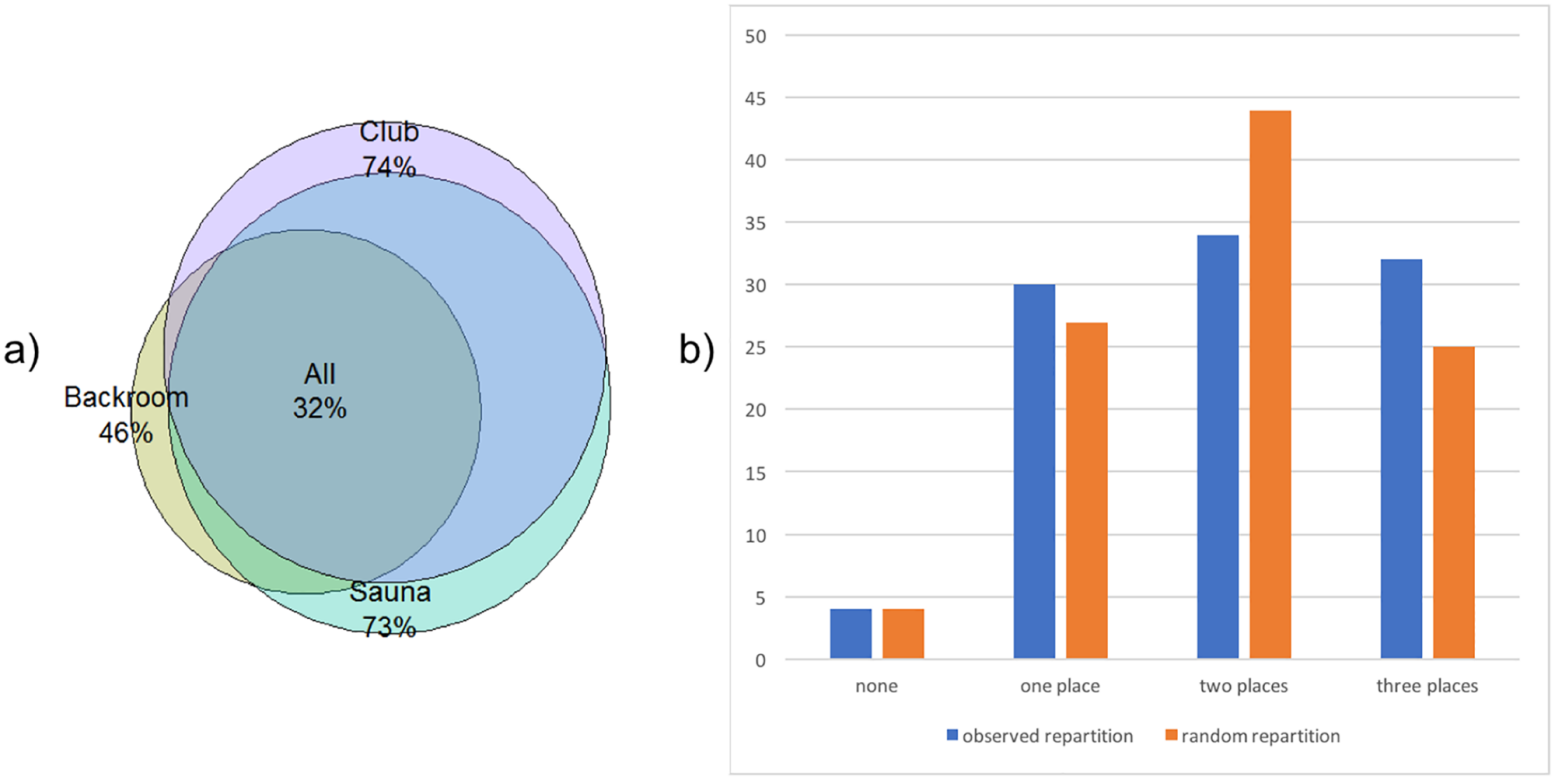
Mixing by venues visited. a) Venue types attended by MSM participating to the PREVAGAY Survey. Percentages are computed with respect to the whole population. b) observed percentage of MSM visiting 0,1, 2 or 3 venue types (PREVAGAY) and expected repartition in the absence of correlation between venues (p<0.001).

PREVAGAY participants had 17 casual partners/year on average (95% CI 16–19), varying according to venues visited (S1 Table). Participants reported visiting saunas, clubs and backrooms in 73% (646/886), 74% (653/886) and 46% (405/886) of the cases (Fig 2). Visiting backrooms was associated with visiting saunas (OR = 1.93, 95%CI 1.42–2.62) and clubs (OR = 2.88, 95%CI 2.08–3.99). On the contrary, visiting clubs and saunas was less than expected by chance (OR = 0.54, 95%CI 0.38–0.78). The HIV prevalence was 19% (95%CI 16–23) in MSM visiting (at least) clubs and 18% (95%CI 15–21) for those visiting (at least) saunas, and increased to 26% (95%CI 22–30) for those visiting (at least) backrooms (p<0.001 for difference with clubs and saunas). Similar, though larger, differences were found in those visiting only one place: prevalence was 12% (95%CI 6–17) for saunas only visitors, 10% (95%CI 5–16) in clubs only visitors and 33% (95%CI 2–64) in backroom only visitors. Backroom visitors were slightly older than in other venues (39.9 years old (95%CI [38.9–40]) vs 37.5 years old (95%CI [36.4–38.5]), p = 0.002), although the difference remained quantitatively small. Multivariable analysis showed that older age, higher number of partners, no condom during anal intercourse and visiting backrooms were independently associated with seropositive status (Table 2).

**Table 2:** Factors associated with positive HIV status in the PREVAGAY study.

Variables	Univariable analysis		Multivariable analysis	
	OR [95% CI]	p	OR [95% CI]	p
**Backroom attendance**	2.98 [2.07–4.30]	<0.001	2.38 [1.52–3.72]	<0.001
**Sauna attendance**	0.98 [0.67–1.45]	0.93	-	
**Club attendance**	1.63 [1.10–2.51]	0.03	-	
**Casual partners number (≥ 3vs. <3)**	2.70 [1.85–3.93]	<0.001	1.73 [1.10–2.74]	0.02
**Condom use during anal sex (No vs. Yes)**	3.10 [2.14–4.49]	<0.001	2.87 [1.89–4.37]	<0.001
**Age**				
≤ 31	1		1	
32–38	2.24 [1.26–3.97]	0.005	1.93 [1.04–3.59]	0.04
38–45	2.49 [1.40–4.40]	0.002	2.06 [1.09–3.87]	0.03
> 45	2.25 [1.27–4.00]	0.005	2.16 [1.15–4.10]	0.02

### Incidence and prevalence with venues visited in different network settings at baseline

**Fig 3:**
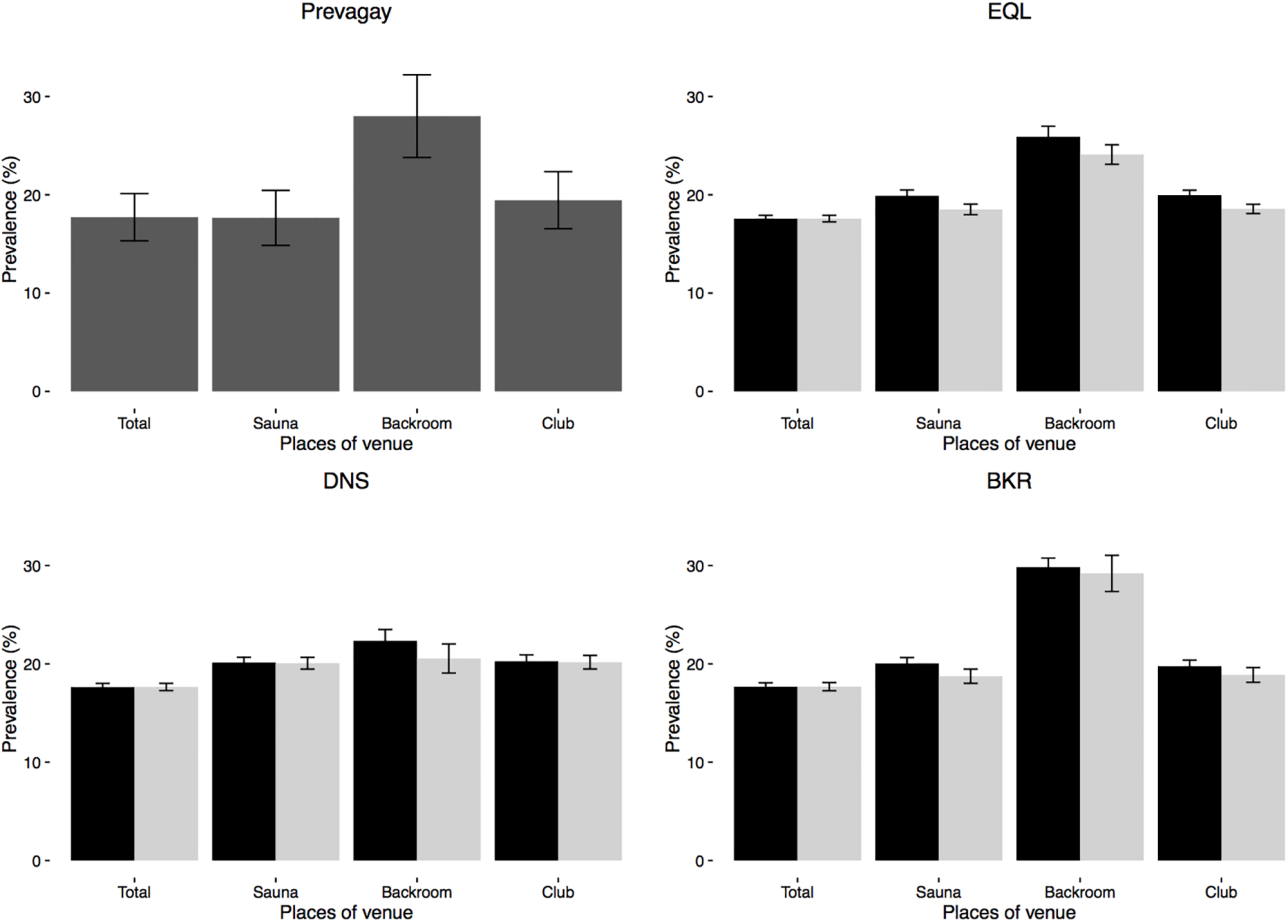
Difference in HIV prevalence in clubs, saunas and backrooms according to assortativity in venues visited. A) PREVAGAY survey. B,C,D) Simulated prevalences for a total of 18% under B) Equal number allocation (EQL); C) equal density allocation (DNS); D) twice more partnerships in Backrooms (BKR). Black bars correspond to the original PREVAGAY data, light gray bars to independence between venues visited (RND/*).

**Fig 4:**
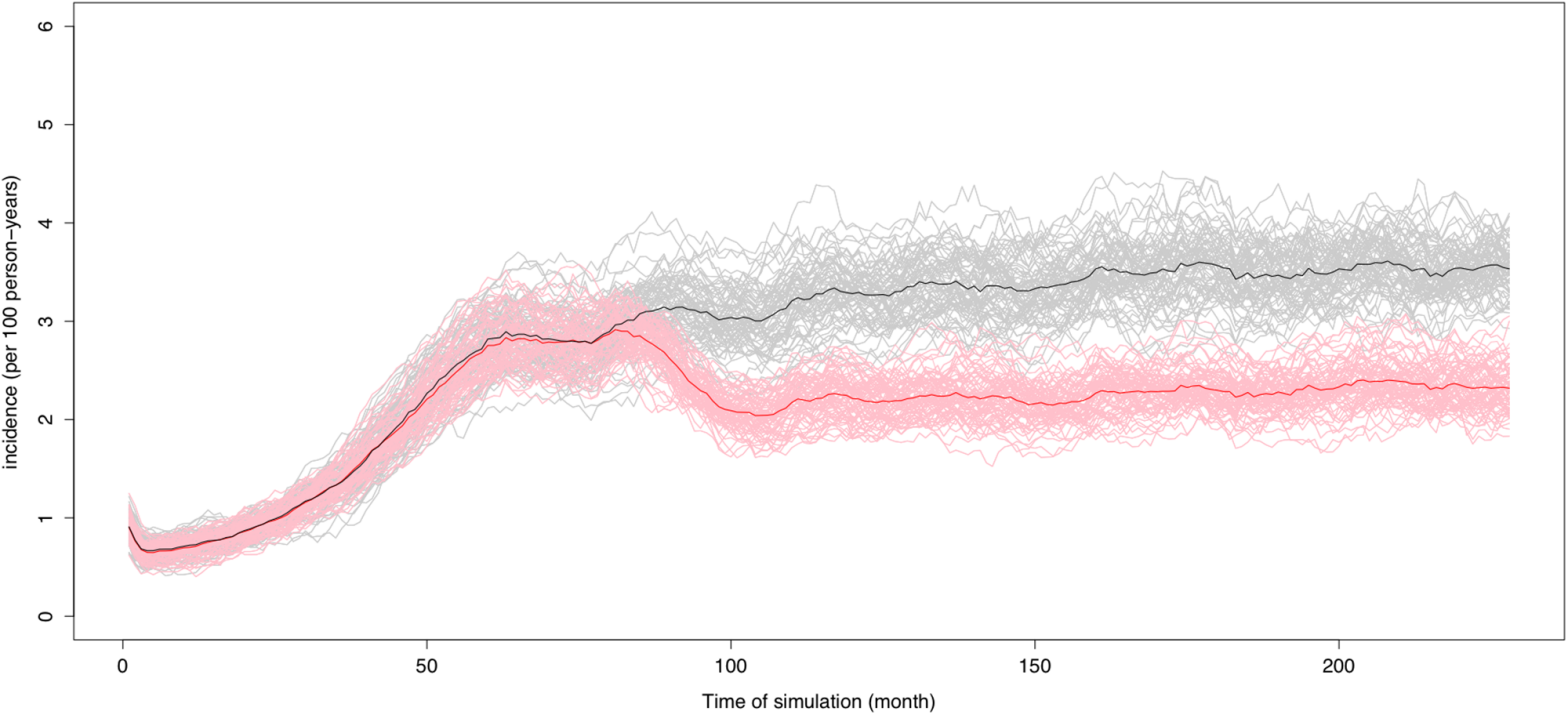
HIV incidence without PrEP (black) and with PrEP (red) in backroom visitors. Results obtained with 20% PrEP coverage in backrooms and the BKR allocation rule.

The simulated sexual networks reproduced the overall number of partners observed in the PREVAGAY study. Allocating new partnerships equally between venues (EQL) or preferentially in backrooms (BKR) yielded numbers by venue types close to that in PREVAGAY. (Table 2). The simulated HIV prevalence was also larger in those attending backrooms than other places. (Fig 3) When the correlation between places visited was removed (RND/* networks), the differences in prevalence were smaller between places. The simulated HIV incidence was between 3 and 4% per year (Fig 4). Allocating more partnerships to backrooms led to an increased circulation of the virus for the same number of contacts: Incidence was 3.08% [2.86–3.32] per year under the DNS allocation and 3.34% [3.05–3.64] per year under BKR allocation, an 8% increase.

The correlation between venues visited led to an increased prevalence by comparison with independent visits to each venues (simulations RND/*). Incidence was 7.5% lower in the absence of correlation between places visited (RND/BKR vs. BKR).

### PrEP effectiveness

Similar results were obtained irrespective of the rules for allocating new partnerships. For the BKR rule, 10% PrEP coverage in MSM used independently of places visited adverted 11.1% (95%CI 10.8–12.5) new infections over 36 months compared to no PrEP. Doubling coverage led to approximately twice more infections adverted over the same period (fraction adverted = 25.4% (95%CI 23.7–27.3)). We found that with the same PrEP coverage, the impact changed according to where the users went: for example, if all the PrEP recipients attended backrooms, 32.2% (95%CI 30.76–33.7) were adverted for an overall coverage of 20% (Table 3). The RND/* scenarios showed that the correlation between places visited reinforced the effect of differential PrEP use according to venues, with 33% more infections adverted in BKR (6.8% = 32.2–25.4, see Table 3) vs RND/BKR(5.1%). Results were similar with the DNS allocation rule.

**Table 3:** Infection adverted by PrEP after 36 months of intervention. Percentages are relative to the baseline scenario including condom use and TasP in seropositive MSM.

Scenario settings	Networks			
10% coverage	BKR	DNS	RND/BKR	RND/DNS
Homogenous	11.1 [10.8–12.5]	11.9 [11.4–12.9]	11.1 [10.8–12.6]	13.1 [11.3–14.1]
Sauna	10.2 [9.8–11.6]	11.2 [1.8–12.6]	11.2 [9.9–12.1]	13.2 [11.4–14.1]
Backroom	15.2 [14.8–16.4]	14.2 [13.8–15.4]	14.6 [13.1–15.9]	13.3 [11.6–14.2]
Club	11.2 [10.6–12.6]	13.2 [12.6–14.1]	11.4 [10.1–12.6]	13.2 [11.6–14.2]
**20% coverage**				
Homogenous	25.4 [23.7–27.3]	24.0 [22.3–25.7]	25.3 [23.7–27.3]	25.9 [23.6–28.1]
Sauna	25.6 [23.8–27.4]	24.6 [22.9–26.3]	27.0 [24.9–29.0]	26.2 [24.4–28.8]
Backroom	32.2 [30.8–33.7]	28.7 [26.9–30.4]	30.4 [28.2–32.6]	26.6 [24.8–29.3]
Club	25.4 [23.0–27.1]	25.7 [24.1–27.4]	27.5 [25.0–30.0]	27.8 [25.1–30.5]

### Direct and indirect effect of PrEP

**Fig 5:**
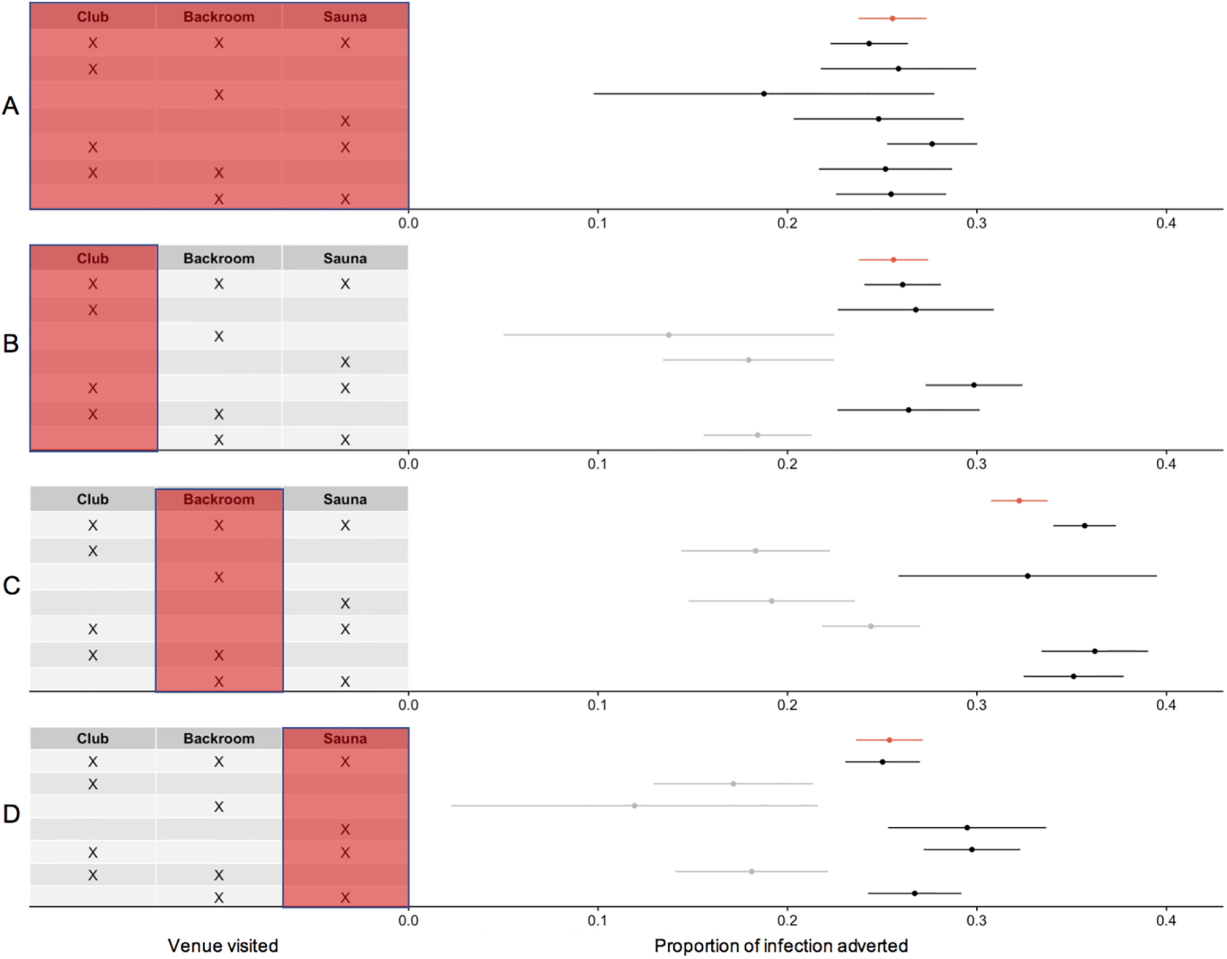
Direct and indirect effect of PrEP coverage: Proportion of infection adverted relative to no PrEP after 36 month. A) homogeneous PrEP use, B) PrEP in saunas, C) PrEP in backrooms, and D) PrEP in clubs. Rows correspond to each pattern of venues visited (Saunas, Backrooms, Clubs), with the proportion of infection adverted. Infection adverted in PrEP users are shown in black (direct effect), and other populations in grey (indirect effect). The average proportion of infection adverted in the whole population is shown in red. Results obtained with 20% PrEP coverage and BKR network.

The proportion of infections adverted was the same in all venue types when PrEP was used independently of places visited (Fig 5A). When users of PrEP went in one place only, the direct effect was the highest when users visited backrooms (see Fig 5B, 5C and 5D). The reduced circulation of HIV in those using PrEP led to indirect protection in non-users, albeit with substantial variation. These indirect effects were the largest when users of PrEP attended backrooms (Fig 5C). In this situation, HIV incidence decreased in those visiting backrooms (from 32.6 to 36.2% reduction) as well as in those not attending backrooms (from 18.3 to 24.4% reduction).

## Discussion

We found that differences in the type of venues visited were associated with differences in HIV prevalence, and could modify the efficiency of pre-exposure prophylaxis prevention depending on who uses PrEP.

### HIV transmission and place-based sexual networks

In the PREVAGAY study, the overall HIV seroprevalence (18%) was less than in two venue types (19% in clubs and 26% in backrooms) and equal to prevalence in sauna. This seemingly counter-intuitive result is possible because the venue types attended are not mutually exclusive. Therefore, overall prevalence need not be a weighted average of that in each venue type. More interestingly, prevalence was the highest in backrooms, though backrooms were the least frequently visited venues. This association between backrooms and increased HIV prevalence remained after adjusting for individual characteristics indicative of risky behavior (no condom and ≥3 partners). Several mechanisms can contribute to the difference in prevalence between venues. Allocating more partnerships in backrooms led to such differences (BKR vs EQL assumptions). Indeed, it is known that sexual behavior can be riskier in backrooms than in other places [19,38,39]. While finding more partners in one place may contribute to the explanation, we also found that the mere correlation between places attended led to such differences, as exemplified with the equal density allocation (DNS rule). Indeed, only by removing correlation between places attended could we get no differences between venue types (see DNS/RND situation). Interestingly, changing venues attended over time between MSMs, for example from “backrooms/clubs” to “saunas” or the converse, led to a levelling of prevalence in all venues, irrespective of how partnerships were allocated. This suggest that both number of partners and correlation between places attended are necessary for such difference to occur. We acknowledge that such differences could also arise from reverse causality, if MSM are more likely to attend backrooms once they are seropositive [40]. More studies documenting change in sexual behavior over time, and before and after HIV seroconversion, would be necessary to precise the relative contributions of these mechanisms. Characteristics of sexual networks, including density, homophily (by ethnicity, sexual behavior), concurrency and temporal structure impact the spread of infectious diseases [24,28,41–48]. Recently, assortativity by race or identity group has been explored in MSM as an explanation to differences in HIV spread in selected subpopulation [48,49]. Yet, substantial heterogeneity remains in HIV spread, calling for more research and data on the association of personal and network related risk factor of being infected by HIV.

### PrEP prescription and impact

PrEP is a promising opportunity to curb HIV incidence in MSM, and thought to be cost-effective [35,45,50–59]. As of today, international guidelines for PrEP prescription use only individual characteristics known to increase HIV transmission (number of sexual partners, condom use) [14]. In the PREVAGAY study, which describes sexually active members of the gay community in Paris, nearly all participants would qualify for PrEP use according to these standards: all individuals had more than one sexual partner/year and most of them infrequently used condoms. Other individual characteristics (ethnicity, drug use, type of anal intercourse) [60] have been studied to help target those at risk of being infected and who would benefit the most from PrEP. Yet, these models did not investigate the role of the sexual network structure, which is indeed a more recent endeavor in models [26,43,49].

We found that heterogeneous PrEP coverage based on venues visited could outperform homogeneous PrEP coverage. This difference of efficacy between types of coverage depended on partnerships allocation but also on correlation between venues visited. This underlines that network structure, here arising from joint attendance to several venue types, could prove useful to understand differences in reported effectiveness, as well as to better target PrEP recipients. Finally, heterogeneous use of PrEP, especially in backrooms, caused large indirect effects in the MSM population. It is a good example on how individual-based prevention such as PrEP can have an effect on subpopulations who do not benefit of this prevention directly. From a practical point of view, this suggests that places attended could be considered in communication plans to promote the use of PrEP. In this work, we considered 20% coverage for PrEP, when reaching 100% of the target population should be the ultimate goal of public health interventions. Indeed, reaching the population of interest will be key to PrEP efficiency. For example, in the United States of America, there had been 125 000 prescriptions of PrEP 5 years after implementation [61], when the target population was 1 200 000 individuals [62]. In France, 3400 individuals had received PrEP 2 years after implementation, out of an estimated target population of “10 000 to 20 000” individuals [63,64]. Slow adoption may lead to wide variation in coverage, depending on the subpopulations of interest [65]. In this respect, our work may help to shed light on differences in PrEP efficiency in the field, even with the same coverage, but the differences would vanish with 100% coverage

### Limitations

We modeled casual sexual partners network according to venue type and on partner allocation in each venue. As such, our model did not account for other individual characteristics that can impact the risk of HIV infection, such as the type of sexual acts or use of prevention, nor on other characteristics affecting new partnerships formation such as age preference or knowledge of serological status. Indeed, individual characteristics will change how partnerships are made within venue types rather than between venues. Additional characteristics could be added to define more precisely the sexual network and the effect of PrEP.

We did not use incidence data to calibrate the model, but only number of partners in the PREVAGAY study and the literature to document the natural history of infection. However, we found that incidence in our simulations were in good agreement with that observed in this population: 3–4% per year in the population (when prevalence reached 18%) vs. 3.8% in the PREVAGAY study [8]. In the same study, 10% of infections were described as “recent”, i.e. less than 6 months old, which again compared with the 8%-9% recent infections in the simulations. Interventions were considered once prevalence had reached 18%. We found that the results regarding PrEP and networks were not substantially changed when starting with other prevalence values in a large range between 10 and 30%.

In this work, the venues under consideration did not include social networks, which are now used for finding partners. However, such popular tools may weakly inform on the structure of the sexual network if they are used by a large fraction of MSM. More specific/selective places provide more information on differences in HIV exposure. Another feature of the PREVAGAY study was to focus on French MSM visiting gay venues in Paris. This population is certainly of importance regarding the use of PrEP, given their characteristics, but is not representative of the whole MSM population. Yet, MSM not attending such venues are expected to be at lower risk of infection and spread. Future works needs to be done to build more realistic network implying more mixing variable including other population considered at lower risk, other kind of partnerships and venues.

As mentioned above, PREVAGAY was a cross-sectional study. This made it impossible to tell if MSM attended, say, backrooms before or after seroconversion, and if they changed habits over time. For example condom use is reportedly decreasing which could affect transmission [66]. Longitudinal surveys are needed to document how sexual behavior change over time. For this reason, we only assessed short term outcomes for PrEP efficicency.

Last, real number of sexual contact made in different venues was unknown in our dataset and led us to arbitrary allocation rules. These were chosen with a view to explore different densities of contact between places, providing a sensitivity analysis to the effect of such rules. Allocating the same density to each subnetwork in a network with correlation at random was the only situation where results of heterogeneous and homogeneous coverage were similar. In other more realistic networks settings, we found similar qualitative effect of different PrEP coverage. Homogeneous coverage was less efficient than heterogeneous coverage and assortativity between venues has a role in PrEP epidemiological efficacy.

In conclusion, sexual network structure, here based on venue type preference for finding partners, may alter the community-level efficacy of individual-based prevention methods such as PrEP. As adherence to most prevention methods is difficult to reach and maintain, taking into account network characteristics in the development of prevention campaigns can be of interest. The main objective of future studies is to better define which subpopulation could have the greatest influence in the epidemic process in MSM sexual network, to evaluate if these people have access to PrEP and, if not, how to make this access possible.

## Supporting information

S1 Table Average number of partners and relative HIV prevalence according to venue type visited. The average number of sexual partners by year was computed for the PREVAGAY study and for each rule of partner allocation according to venue type. *Relative to overall prevalence (18%).
(DOCX)

S1 Appendix Data file containing PREVAGAY variable used in the analysis. (CSV)

S2 Appendix Detailed information on the transmission model used in the simulations. (DOCX)
