# Supplementary material for: HIV transmission and pre-exposure prophylaxis in a high risk MSM population: A simulation study of location-based selection of sexual partners
Source: PLoS One. 2017 Nov 30;12(11):e0189002. doi: 10.1371/journal.pone.0189002 (PMC5708822; doi:10.1371/journal.pone.0189002)
Supplement: S1 Table — The average number of sexual partners by year was computed for the PREVAGAY study and for each rule of partner allocation according to venue type. *Relative to overall prevalence (18%). (DOCX) [file pone.0189002.s001.docx]

|  | Average number of partners [95%CI] | | | | Relative HIV prevalence* [95%CI] | | |
| --- | --- | --- | --- | --- | --- | --- | --- |
| Network | Overall | Sauna | Backroom | Club | Sauna | Backroom | Club |
| PREVAGAY | 17 [16-19] | 20 [19-21] | 24 [23-26] | 18 [17-20] | 1 [0.93-1.07] | 1.21 [1.13-1.29] | 1.08 [1.02-1.15] |
| EQL | 17 | 18 | 24 | 19 | 1.10 | 1.18 | 1.09 |
| BKR | 17 | 18 | 26 | 19 | 1.10 | 1.25 | 1.12 |
| DNS | 17 | 18 | 21 | 19 | 1.12 | 1.12 | 1.13 |

**S1 Table. Average number of partners and relative HIV prevalence according to venue type visited.** The average number of sexual partners by year was computed for the PREVAGAY study and for each rule of partner allocation according to venue type. *Relative to overall prevalence (18%).
