# Supplementary material for: HIV transmission and pre-exposure prophylaxis in a high risk MSM population: A simulation study of location-based selection of sexual partners
Source: PLoS One. 2017 Nov 30;12(11):e0189002. doi: 10.1371/journal.pone.0189002 (PMC5708822; doi:10.1371/journal.pone.0189002)
Supplement: S2 Appendix — (DOCX) [file pone.0189002.s003.docx]

**Supplementary appendix**

As describe in the full paper, models used are agent-based microsimulation models describing a network of sexual contact evolving within time. Statistics need to build the three subnetwork are given in the article. This supplementary appendix describes the mathematical model of transmission of HIV through the networks. At each time steps of one day, HIV can be transmitted to an individual to another if, at this time, a sexual contact (or edge) exists between an HIV infected individual and a non-HIV infected individual. The probability of being infected during a sexual contact is highly correlated with viral load of the source which depends on treatment efficacy and evolution of the disease (with or without treatment). Parameters modifying the viral load might change monthly and are detailed in this appendix. The other variable that influence transmission are personal prevention intake such as PreP and condom use. Their influence on transmission probabilities are also detailed in this appendix.

**A Probability of infection without prevention policies**

The probability (β) of infection for each sexual relation is dependent of the viral load of the source and the sex of each partners (here only men). (1,2) :

β*=2·45log10(V1/V0)* * β_0_  [1] (2·45 (95% CI 1.85–3.26)

where β_0_ is the probability of being infected depending on the type of sexual relation (here MSM) for a viral load of 10^4.5

The probability of being infected during a sexual contact is:

P=1-(1- β)^n^ [2]

Where n is the average number of sexual relation by time step with this partner.

**B Intervention:**

The probability of being infected at each time steps is:

P’=1-(1- (1-P_intervention_)* β)^n^ [3]

where

P_intervention_ =P_condom_ +(1- P_condom_)* P_prep_ [4]

1. **Condom use: P_condom_**

Efficacy of condom depends on its use (« always », « often », « never ») as describe in (12).

1. **Pre-Exposure Prophylaxis (PreP): P_prep_**

There are few data on PreP use in real life. We consider PreP efficacy using results of iPrEx studies (13) (14) (15).

**3) Defining the transmitter population**

All infected individual can be a transmitter, except those diagnosed for AIDS. We consider that AIDS diagnosis is done in the month following the date individual reach this stage of infection.

| **Name** | **Description** | **Value** | **References** |
| --- | --- | --- | --- |
| β | Probability of being infected at each sexual relation | *see equation 1* | (1,2) |
| V1 | Mean viral load | 20-10^8^ | (1,2) |
| V0 | First viral load | 10^4.5^ | (1,2) |
| β0 | Probability of being infected with a viral load of 10^4.5 copies/ml | 0,01 | (1,2) |
| n | Number of sexual act | 1 |  |
| P_condom_ | Condom efficacy depending on its use frequency | Always: 0.90-0.95  Often: 0.5-0.80  Never:0 | (3) |
| P_prep_ | PreP efficacy | 0.44 | (4–6) |

**Table S1: Variable conditioning the probability of transmission**

**C Viral Load evolution (VL) (table S2)**

1. **Natural evolution of the viral load**
2. *3 first months:*

The viral load is increasing quickly before decreasing to the chronical viral load value at the end of the third month of infection (3) (4).

1. *Chronical part of the disease: (9,10)*

There is a wide variety of data and publication on the natural evolution of the viral load. Our model is based on Hubert and al. paper (11) . These French data are close to variation found in other series (12) .After the first period of infection (primo-infection) we consider that viral load is following a log-normal distribution (4, 0.75). Each infected individual has a viral load chosen at random on this law. Three groups of wild evolution of the VL are defined as shown in table S2.

| **Name** | **Description** | **Value** | **references** |
| --- | --- | --- | --- |
| VL.wild | Viral load defined monthly without any treatment | month 1: log(10^7^)  month 2: log(10^6^)  month 3: (10^5^)  month 4: x  month 5 to the end of follow-up:   - Standard progression group   - time 6 to 18: x   - time 19 to the end of follow-up:   x+(t-18)*0.10/12   - Slow progression group   - time 6 to 48: x   - time 49 to the end of follow-up: x-(t-48)*0.03/12 - Rapid evolution group:   - time 6 to 18: x   - time 19 to the end of follow-up:   x+(t-18)*0.25/12  VL max= log(10^8^) | (2,13–15)  (5,9–11) |
| x | Initial value of the viral load after the three first month of infection | Chosen at random on a log normal distribution (4,0.75) | (11,5) |
| VL.prep | Viral load under PreP | VL.wild-2 | (16,17) |
| VL. treatment | Viral load of individuals under treatment depending on the wild viral load and treatment efficacy | Good responders: diminution of 2 log the first month then diminution of 0.5 log monthly.  Partial responders: diminution of 2 log the first month then stabilization of the viral load until the end of the follow-up.  Not responders: VL.treatment = VL.wild  CV.min= log(20) | (18) |

**Table S2: Variables involve in the definition of the viral load value.**

1. **Evolution of viral load under PreP**

It is difficult to estimate PreP influence on VL in case of infection under this treatment. Survey on monkeys suggest that PreP makes the viral load decreased of 2 log (13–15). We consider that PreP is stopped after a positive screening test which is performed every 3 months in this population under PreP.

PreP is stopped if individual is diagnosed HIV positive. Treatment start the month after.

1. **Evolution of viral load under treatment:**

We decide to take into account three situations (18):

- Good responders: diminution of 2 log the first month then diminution of 0.5 log monthly to <20copies/ml.
- Partial responders: diminution of 2 log the first month then stabilization of the viral load until the end of the follow-up.
- Not responders: VL.treatment = VL.wild

Probabilities of being a good, a partial or a non-responder to treatment are as follow : (18)

- good responder= 0.83
- Partial responder: 0.08
- Non-responder: 0.09

**C. CD4 blood count:**

We considered that VL and CD4 count are independent. (19).

Four evolution groups has been established with different initial value and decreasing slope as described in (9) (640, 670, 768 et 785 CD4/ml on average at the beginning. CD4 count decreased by 249,89,87,73 CD4/mm^3^ /year respectively)

Under treatment CD4 increased above 200/mm2 within a year, under partial efficient treatment CD4 value remained constant.

**D. Probability of being under treatment**

AIDS event will necessarily make the treatment start. Treatment is started within the month of diagnosis of HIV infection, following Treatment as Prevention (TasP) guidelines.

**E. Probability of an AIDS event without treatment (table S3)**

This probability depended on VL, CD4 count and age (7,8). This probability follows an exponential law:

**λ** _AIDS.1_= 1⁄4 exp{-3.55 + [-0.21*$\surd$(CD4)] + 0.71 (log VL) + 0.024(Age)}

P_AIDS.1_ =[1 - exp(-1/12* **λ** _SIDA.1_)]

**F) Probability of an AIDS event without treatment**

This probability follow a Poisson law with the incidence of an AIDS event as a parameter. This incidence depends of CD4 count and of the viral load if CD4 where above 250/mm3 (12).

**G) HIV Diagnostic Testing for HIV**

Testing for HIV is done yearly for the whole population and every three months for people under PreP.

| **Name** | **Description** | **Value** | **references** |
| --- | --- | --- | --- |
| **λ** _AIDS.1_ | Parameter determining the probability of an AIDS event at each time step | 1⁄4 exp{-3.55 + [-0.21*√(CD4)] + 0.71 (log VL) + 0.024(Age)} | (7,8) |
| P_AIDS.1_ | Probability of an AIDS event without treatment at each time step |  | (7,8) |
| **λ** _AIDS.2_ | Probability of an AIDS event under treatment. | if CD4>250:   - If VL<1000: 5/ (739*12) - If   VL>1000: 9/ (295*12)  If 100<CD4<=250: 12/ (140*12)  If CD4<=100: 10/ (44*12) | (12,9) |
| P_AIDS.2_ | Probability of an AIDS event at each time step | Exp( **λ** _AIDS.2_ (e^t^-1) | (12,9) |

**Table S3: Variables defining the probability of an AIDS event**

**Bibliography**

1. Røttingen J-A, Garnett GP. The epidemiological and control implications of HIV transmission probabilities within partnerships. Sex Transm Dis. 2002 Dec;29(12):818–27.

2. Wilson DP, Law MG, Grulich AE, Cooper DA, Kaldor JM. Relation between HIV viral load and infectiousness: a model-based analysis. Lancet. 2008 Jul 26;372(9635):314–20.

3. Weller S, Davis K. Condom effectiveness in reducing heterosexual HIV transmission. Cochrane Database Syst Rev. 2001;(3):CD003255.

4. Thigpen MC, Kebaabetswe PM, Paxton LA, Smith DK, Rose CE, Segolodi TM, et al. Antiretroviral Preexposure Prophylaxis for Heterosexual HIV Transmission in Botswana. The New England journal of medicine [Internet]. 2012 Jul 11 [cited 2012 Jul 23]; Available from: http://www.ncbi.nlm.nih.gov/pubmed/22784038

5. Baeten JM, Donnell D, Ndase P, Mugo NR, Campbell JD, Wangisi J, et al. Antiretroviral Prophylaxis for HIV Prevention in Heterosexual Men and Women. The New England journal of medicine. 2012 Jul 11 [cited 2012 Jul 23]; Available from: http://www.ncbi.nlm.nih.gov/pubmed/22784037

6. Grant RM, Lama JR, Anderson PL, McMahan V, Liu AY, Vargas L, et al. Preexposure Chemoprophylaxis for HIV Prevention in Men Who Have Sex with Men. New England Journal of Medicine. 2010 Dec 30;363(27):2587–99.

7. Phillips A, Pezzotti P. Short-term risk of AIDS according to current CD4 cell count and viral load in antiretroviral drug-naive individuals and those treated in the monotherapy era. AIDS. 2004 Jan 2;18(1):51–8.

8. Phillips AN, Lundgren JD. The CD4 lymphocyte count and risk of clinical progression. Curr Opin HIV AIDS. 2006 Jan;1(1):43–9.

9. Lyles RH, Muñoz A, Yamashita TE, Bazmi H, Detels R, Rinaldo CR, et al. Natural history of human immunodeficiency virus type 1 viremia after seroconversion and proximal to AIDS in a large cohort of homosexual men. Multicenter AIDS Cohort Study. J Infect Dis. 2000 Mar;181(3):872–80.

10. Lyles CM, Dorrucci M, Vlahov D, Pezzotti P, Angarano G, Sinicco A, et al. Longitudinal Human Immunodeficiency Virus Type 1 Load in the Italian Seroconversion Study: Correlates and Temporal Trends of Virus Load. J Infect Dis. 1999 Jan 10;180(4):1018–24.

11. Hubert JB, Burgard M, Dussaix E, Tamalet C, Deveau C, Le Chenadec J, et al. Natural history of serum HIV-1 RNA levels in 330 patients with a known date of infection. The SEROCO Study Group. AIDS. 2000 Jan 28;14(2):123–31.

12. Tarwater PM, Gallant JE, Mellors JW, Gore ME, Phair JP, Detels R, et al. Prognostic value of plasma HIV RNA among highly active antiretroviral therapy users. AIDS. 2004 Dec 3;18(18):2419–23.

13. Pilcher CD, Tien HC, Eron JJ, Vernazza PL, Leu S-Y, Stewart PW, et al. Brief but Efficient: Acute HIV Infection and the Sexual Transmission of HIV. J Infect Dis. 2004 May 15;189(10):1785–92.

14. Pilcher CD, Joaki G, Hoffman IF, Martinson FEA, Mapanje C, Stewart PW, et al. Amplified transmission of HIV-1: comparison of HIV-1 concentrations in semen and blood during acute and chronic infection. AIDS. 2007 Aug 20;21(13):1723–30.

15. Kaufmann GR, Cunningham P, Kelleher AD, Zaunders J, Carr A, Vizzard J, et al. Patterns of Viral Dynamics During Primary Human Immunodeficiency Virus Type 1 Infection. J Infect Dis. 1998 Jan 12;178(6):1812–5.

16. García-Lerma JG, Otten RA, Qari SH, Jackson E, Cong M, Masciotra S, et al. Prevention of Rectal SHIV Transmission in Macaques by Daily or Intermittent Prophylaxis with Emtricitabine and Tenofovir. PLoS Med [Internet]. 2008 Feb [cited 2012 Jul 23];5(2). Available from: http://www.ncbi.nlm.nih.gov/pmc/articles/PMC2225435/

17. Supervie V, García-Lerma JG, Heneine W, Blower S. HIV, transmitted drug resistance, and the paradox of preexposure prophylaxis. Proc Natl Acad Sci U S A. 2010 Jul 6;107(27):12381–6.

18. Madge S, Smith CJ, Lampe F, Sabin CA, Youle M, Johnson MA, et al. An audit of viral load in one clinical population to describe features of viraemic patients on antiretroviral therapy. HIV Med. 2008 Apr;9(4):208–13.

19. Rodríguez B, Sethi AK, Cheruvu VK, Mackay W, Bosch RJ, Kitahata M, et al. Predictive value of plasma HIV RNA level on rate of CD4 T-cell decline in untreated HIV infection. JAMA. 2006 Sep 27;296(12):1498–506.
